# Immobilization of Interfacial Activated *Candida rugosa* Lipase Onto Magnetic Chitosan Using Dialdehyde Cellulose as Cross-Linking Agent

**DOI:** 10.3389/fbioe.2022.946117

**Published:** 2022-07-18

**Authors:** Shushu Wang, Shan Li, Runtang Liu, Wei Zhang, Huajin Xu, Yi Hu

**Affiliations:** State Key Laboratory of Materials-Oriented Chemical Engineering, School of Pharmaceutical Sciences, Nanjing Tech University, Nanjing, China

**Keywords:** *Candida rugosa* lipase, lipase immobilization, dialdehyde cellulose, magnetic nanoparticles, interface activation

## Abstract

*Candida* *rugosa* lipase (CRL) was activated with surfactants (sodium dodecyl sulfate [SDS]) and covalently immobilized onto a nanocomposite (Fe_3_O_4_-CS-DAC) fabricated by combining magnetic nanoparticles Fe_3_O_4_ with chitosan (CS) using polysaccharide macromolecule dialdehyde cellulose (DAC) as the cross-linking agent. Fourier transform infrared spectroscopy, transmission electron microscope, thermogravimetric analysis, and X-ray diffraction characterizations confirmed that the organic–inorganic nanocomposite support modified by DAC was successfully prepared. Enzymology experiments confirmed that high enzyme loading (60.9 mg/g) and 1.7 times specific enzyme activity could be obtained under the optimal immobilization conditions. The stability and reusability of immobilized CRL (Fe_3_O_4_-CS-DAC-SDS-CRL) were significantly improved simultaneously. Circular dichroism analysis revealed that the active conformation of immobilized CRL was maintained well. Results demonstrated that the inorganic–organic nanocomposite modified by carbohydrate polymer derivatives could be used as an ideal support for enzyme immobilization.

## Introduction

Enzymes are a class of biological macromolecules with catalytic activity and specific spatial conformation in organisms, and they are widely used in chemical industry, medicine, food, and energy (Ismail et al., 2021; Sherawat et al., 2021). *Candida rugosa* lipase (CRL) is a lipase with important application value, which has high activity and specificity and can be widely used in chemical, cosmetic, food, and other applications (Barriuso et al., 2016; Vanleeuw et al., 2019). However, free CRL is very susceptible to external conditions and has the disadvantages of poor stability and difficult to reuse, which limit the application of CRL in large-scale production.

Immobilization can improve the operational stabilization and activity of enzymes and sometimes can even modulate the selectivity of enzymes by immobilization on functional supports (Mateo et al., 2007; Rodrigues et al., 2011; Barbosa et al., 2013). At the same time, the immobilized enzymes can be easily separated from products, which reduce the protein contamination in the product and are conducive to the continuity of production (Xiang et al., 2017; Liang et al., 2020). In recent years, organic–inorganic composites based on Fe_3_O_4_ and natural organic polymers with the characteristics of excellent biocompatibility, high mechanical stability, and good recyclability have received more and more attention and obtained good applications in enzyme immobilization (Faraji et al., 2021; Matveeva et al., 2021). Chitosan (CS) is an inexpensive and available natural polysaccharide. Owing to its excellent biocompatibility, nontoxicity, and good biodegradability, CS has been valued in the research of modification of supports for enzyme immobilization (Nunes et al., 2021). Wang et al. (2015) fabricated composites (GO-CS-Fe_3_O_4_) that consisted of magnetic graphene oxide–chitosan (GO-CS) and magnetite nanoparticles (Fe_3_O_4_) and that was activated with glutaraldehyde (GA) to immobilize CRL. The immobilized CRL exhibited excellent properties such as improved stability, pH, and temperature tolerance. However, the activity recovery was only 60.2% with a moderate enzyme loading obtained (48.5 mg/g). Ziegler-Borowska et al. (2017) coated CS and rat tail collagen onto the surface of magnetic nanoparticles Fe_3_O_4_, and then the support was condensed with CRL by the function of EDC/NHS to prepare the immobilized CRL. The activity recovery rate of the immobilized CRL was as high as 112.0%, and the thermal stability was also enhanced, while the support had a low enzyme loading (27.0 mg/g). Hence, organic–inorganic nanocomposites are still challenging to greatly improve the enzymatic properties of activity, stability, and reusability and obtain high enzyme loading at the same time.

In general, GA is widely used to immobilize enzymes by way of covalent cross-linking. However, this immobilization process is relatively violent, and its high toxicity often results in the loss of enzyme activity (Leung et al., 2001). Starch and cellulose are the most common natural polysaccharides, which are inexpensive, available, and biocompatible. Dialdehyde starch (DAS) and dialdehyde cellulose (DAC) are easy to be prepared by selective oxidation of starch and cellulose. Owing to the abundant aldehyde and hydroxyl functional groups, DAS and DAC have been successfully used as new efficient covalent cross-linking reagents in the process of enzyme immobilization (Sharma et al., 2014; Ruan et al., 2018; Tamaddon et al., 2019). Qiao et al. (2022) prepared magnetic dialdehyde cellulose and used it for the immobilization of bacterial laccase. The pH and inhibitor tolerance, activity, and stability of immobilized laccase were obviously improved. In recent times, a study from our group has verified that DAS could be firmly combined with laccase onto Fe_3_O_4_ modified by amino-functionalized ionic liquid to stabilize its three-dimensional conformation; compared with those cross-linked by GA, the enzymatic properties of laccase were improved by a larger extent (Qiu et al., 2021).

The active center of lipase is usually covered by a “lid” structure (Verger et al., 1997; Schmid et al., 1998). In addition to the strategy of immobilizing lipase on a hydrophobic carrier to open its lid structure and stabilize its active conformation, addition of surfactants is also a commonly used method to activate lipase by stabilization of the open form of lipase (Lopez et al., 2002; Manoel et al., 2015; De Oliveira et al., 2018). Calvo et al. (1996) studied the effect of SDS and Triton X-100 surfactants on the hydrolysis activity of isoenzymes (CRL-A and CRL-B). Compared with nonionic-type surfactant Triton X-100, anionic surfactant SDS showed less toxic effects and had better stability to lipases (Calvo et al., 1996). Betancor et al. (2006) found that the addition of surfactants could further improve the enzymatic properties of CRL covalently immobilized on aminated Fe_3_O_4_ support by restraining the aggregation of lipase and decreasing the formation of dimer of lipase to maintain enzymatic activity. In the previous research of our laboratory, surfactants improved the activity and stability of CRL encapsulated in sol–gel materials, and the interface-activated immobilized CRL was successfully applied to catalyze the synthesis of vitamin E succinate (Hu et al., 2013).

Herein, this study intends to expand the support design and enzyme immobilization methods developed by our research group in the early stage, using biological macromolecule DAC as the cross-linking agent and surfactant as the interface activator to prepare an immobilized CRL based on magnetic CS nanocomposites. Under the premise of high enzyme loading, the activity, stability, and reusability of the enzymes will be greatly improved; thus, a new method of highly efficient enzyme immobilization will be developed.

## Materials and Methods

### Materials

CRL (500.0 U/g) was purchased from Sigma-Aldrich; FeCl_3_·6H_2_O (99.0%), FeCl_2_·4H_2_O (99.0%), microcrystalline cellulose (>90 um), chitosan (78% degree of deacetylation), sodium dodecyl sulfate (>88.0%), glutaraldehyde (70% in water), sodium periodate (>98%), Triton X-100 (>98.0%), and Tween-80 (AR) were purchased from Sinopharm Chemical Reagent Co., Ltd. Other chemicals were of analytical grade.

### Characterizations

Transmission electron microscopy (TEM) was carried out on a Tecnai G2 F20 (USA) and Talos F200x (USA). Fourier transform infrared (FT-IR) spectroscopy analysis was carried out using a Nicolet iS5 FT-IR spectrometer (Thermo Nicolet IS5, USA) in a wavelength range of 450–4,000 cm^−1^. Thermogravimetric analysis was conducted on a TG209F3 instrument by heating from 20 to 800°C under N_2_ with a heating rate of 20°C/min. X-ray diffraction (XRD) analysis was performed on a Bruker D8 Advance instrument (Germany). The circular dichroism characterization was performed on the J-1500CD spectrometer (Japan), the scanning speed was 30 nm/min, and the wavelength range was 190–260 nm.

### Synthesis of Fe_3_O_4_ and Dialdehyde Cellulose

The synthesis of Fe_3_O_4_ nanoparticles was referred to the previously reported literature with a slight modification (Suo et al., 2018). The specific process was as follows: FeCl_3_·6H_2_O (5.5 g) and FeCl_2_·4H_2_O (2.1 g) were dissolved in purified water (100 ml) under magnetic stirring, and the reaction solution was heated to 70°C under nitrogen atmosphere. Then, ammonium hydroxide (28%) was used to adjust the pH until the solution was reddish brown, and the temperature was raised to 80°C for 1.5 h. At last, Fe_3_O_4_ nanoparticles were separated by a magnet after cooling to room temperature and washed with purified water until neutral.

DAC was prepared using a method similar to the conventional procedure (Varma et al., 2002). First, 150 ml of distilled water was added to a light-proof round bottom flask, and 1% hydrochloric acid was used to adjust the pH to 3. Then cellulose (3.0 g) and sodium periodate (6.0 g) were added into the flask and stirred at 35°C for 3 h. Next, 2 ml ethylene glycol was added into the flask and kept for 2 h to remove excess sodium periodate. At last, the resulting suspension was suction filtered to obtain a filter cake. The filter cake (DAC) was washed with distilled water several times and dried at 50°C.

### Preparation of Supports

The synthesis of supports Fe_3_O_4_-CS, Fe_3_O_4_-CS-GA, and Fe_3_O_4_-CS-DAC were referred to the previously reported literature with a minor modification (Kim et al., 2017; Zhao et al., 2018).

First, CS (0.3 g) was dispersed in 50 ml acetic acid solution (1%, v/v) and sonicated for 15 min at room temperature. At the same time, Fe_3_O_4_ (1.5 g) was added to 50 ml acetic acid solution (1%, v/v) and stirred to obtain Fe_3_O_4_ acetic acid suspension. Then, the Fe_3_O_4_ acetic acid suspension was slowly dropped into the CS/acetic acid solution and stirred for 30 min at room temperature. Next, NaOH (1%, v/v) was added to adjust the pH to neutral. At last, Fe_3_O_4_-CS nanocomposites was separated by the magnet and washed with pure water several times. The resulting support was denoted as Fe_3_O_4_-CS.

At first, CS (0.2 g) was dispersed in 50 ml acetic acid solution (1%, v/v) and sonicated for 15 min. Then, GA (2 ml) or DAC (0.5 g) was added to the CS/acetic acid solution, and NaOH (1%, v/v) was added to adjust the pH to 7.0. Next, the system was stirred at 30°C for 4 h. At last, CS-GA or CS-DAC was filtered and washed with pure water several times. Fe_3_O_4_ (1.0 g) was dispersed to 25 ml acetic acid solution (1%, v/v), and the 1.5 g CS-GA or CS-DAC was added to this suspension. Next, the system was stirred for 30 min at room temperature. Then, NaOH (1%, v/v) was added to adjust the pH to neutral. At last, nanocomposites was separated by a magnet and washed with pure water several times. The resulting supports were denoted as Fe_3_O_4_-CS-GA or Fe_3_O_4_-CS-DAC.

### *Candida rugosa* Lipase Immobilization

Anionic surface-active (SDS) and nonionic surface-active agents (Tween 80, Triton X-100) were selected to investigate the effects of different types of surfactants on the immobilization of CRL. Different surfactants (0.02 mol) were added to the phosphate buffer solution (PBS) (5 ml, pH 7.0, 0.2 M). Then, CRL (40 mg) was added to the solution and stirred at 10°C for 2 h. Next, Fe_3_O_4_-CS-DAC (0.1 g) was dispersed in the PBS (5 ml, pH7.0, 0.2 M), and the system was stirred at 30°C for 4 h. At last, the prepared immobilized CRL was separated by the magnet and washed several times with PBS (0.2 M, pH7.0). The prepared immobilized CRL was designed as Fe_3_O_4_-CS-DAC-SDS-CRL. Other supports were referred to the above method to immobilize CRL and named as Fe_3_O_4_-CS-SDS-CRL and Fe_3_O_4_-CS-GA-SDS-CRL. The support (Fe_3_O_4_-CS, Fe_3_O_4_-CS-GA or Fe_3_O_4_-CS-DAC) with CRL without surfactants were immobilized according to the above method, and the prepared immobilized CRL was designed as Fe_3_O_4_-CS-CRL, Fe_3_O_4_-CS-GA-CRL, or Fe_3_O_4_-CS-DAC-CRL (Supplementary Figures S1–S3).

### Activity Experiment

**FIGURE 1:**
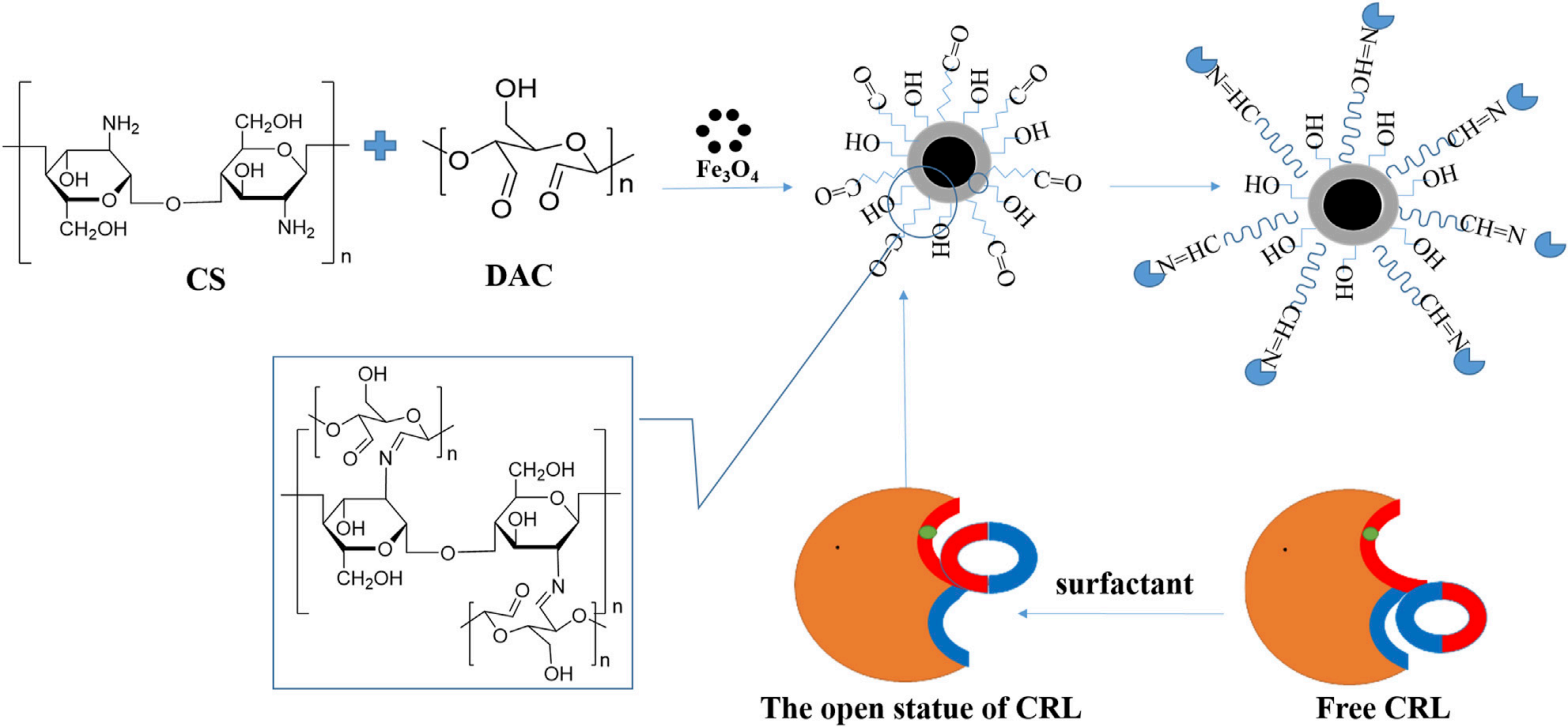
Schematic diagram of preparation of immobilized support and immobilized *Candida rugosa* lipase (CRL).

The preparation of immobilized support and immobilized CRL is shown in Figure 1. The BCA method was used to detect the protein content in the supernatant, and the following formula was used to calculate CRL loading and immobilization efficiency (Walker et al., 1994). Each result was measured three times, and the average value was taken.

$$\mathrm{CRL}\;\mathrm{loading}\left(\text{mg}/g\right)=\left(C_{i}*m_{i}-C_{f}*V_{f}\right)/m_{f}$$ (1)

$$\mathrm{Immobilization}\;\mathrm{efficiency}\left(\text{\%}\right)=\left[\left(C_{i}*\;m_{i}-C_{f}*V_{f}\right)/C_{i}\text{*}m_{i}\right]\times 100\%$$ (2)

where $C_{i}$ is the initial protein concentration in CRL before immobilization (mg/g) , $C_{f}$ is the final protein concentration in supernatant and washing liquid after immobilization (mg/ml) , $m_{i}$ is the mass of CRL added to the buffer solution (g) , $V_{f}$ is the total volume of the supernatant and washing liquid (ml) , and $m_{f}$ is the mass of the support (g) .

### *Candida rugosa* Lipase Activity Assay

The activity of free and immobilized CRL were determined using the olive oil hydrolysis method (Monier et al., 2010; Li et al., 2015). Olive oil (50 ml) and gum arabic solution (50 ml, 14%, w/v) were mixed into a substrate solution. Thereafter, the substrate solution (5 ml) and PBS (5 ml, 0.2 M) were put into an Erlenmeyer flask and incubated at their optimum temperature and pH for 10 min. Then, free or immobilized CRL were added to the substrate solution and reacted 10 min. At last, acetone/ethanol (10 ml, 1/1, v/v) was added to terminate the hydrolysis reaction. The NaOH solution (0.05 mol/L) was used to titrate the fatty acid produced by hydrolysis. One unite (1 U) was defined as the amount of enzymes required to catalyze the hydrolysis of 1 μmol of acid per minute. The following formulas were used for the activity recovery rate, apparent activity, and specific activity of the enzymes. Each result was measured three times, and the average value was taken.

$$\mathrm{Expressed}\;\mathrm{activity}\left(U/g\right)=\left(\left(V-V_{0}\right)\times C_{\mathrm{NaOH}}\right)/\left(M_{1}\times T\right)\times 10^{6}$$ (3)

$$\mathrm{Specific}\;\mathrm{activity}\left(U/g\right)=\mathrm{Expressed}\;\mathrm{activity}/M_{2}$$ (4)

where V is the volume (ml) of NaOH consumed by free or immobilized CRL hydrolyzed substrate to generate fatty acid, $V_{0}$ is the volume of NaOH consumed by the blank (L) , $C_{\mathrm{NaOH}}$ si the concentration of NaOH (mol/L) , T is the reaction time (min) , $M_{1}$ is the mass of free or immobilized CRL (g) , and $M_{2}$ is the mass of protein in the free or immobilized CRL (g) .

### Optimal pH and Temperature

To study optimal pH conditions for the activity assay, free and immobilized CRL activity was measured under different pH values (pH6.0–8.0) at 40°C. The optimum temperature values were obtained by examining the activity of free and immobilized CRL at 30–50°C under respective optimal pH conditions. With the relative activity as an index, the maximum activity was defined as 100%.

### Measurement of Enzyme Stability

In order to determine the thermal stability, the free and immobilized CRL were stored at 55°C for a period of time, and then the enzyme activity was measured every 30 min. The free and immobilized CRL were stored at 4°C in the refrigerator for 1 month to determine the storage stability, and then the enzyme activity was checked every 5 days. The enzyme activity was measured under the optimum conditions, and the initial enzyme activity was defined as 100%.

### Kinetic Parameters of Immobilized Enzyme

Kinetic parameters are important indexes for evaluating immobilized CRL. We adopted Michaelis–Menten model to evaluate the kinetic properties of free CRL and immobilized CRL, the same amount of immobilized CRL were added to triacetin emulsification solution and reacted for 3 min at respective optimum pH and temperature. Calculate the Km and Vmax values through lin-burkpolt plotting according to the following formula:

$$1/V=K_{m}/\left(V_{\max}\left[\text{S}\right]\right)+1/V_{\max}$$ (5)

where [S] is the substrate concentration (mg/ml) and V is the reaction rate (mg/ml min) .

### Reusability

The reusability of immobilized enzymes were investigated through the determination of the residual in the olive oil hydrolysis under their respective optimal hydrolysis conditions. After completing a reaction cycle, the immobilized enzymes were separated by magnetic separation and washed with PBS. Then the recovered immobilized enzymes was put back into fresh reaction medium for the next cycle, and their activity was tested after each cycle.

## Results and Discussion

### Analysis of Characterizations

**FIGURE 2:**
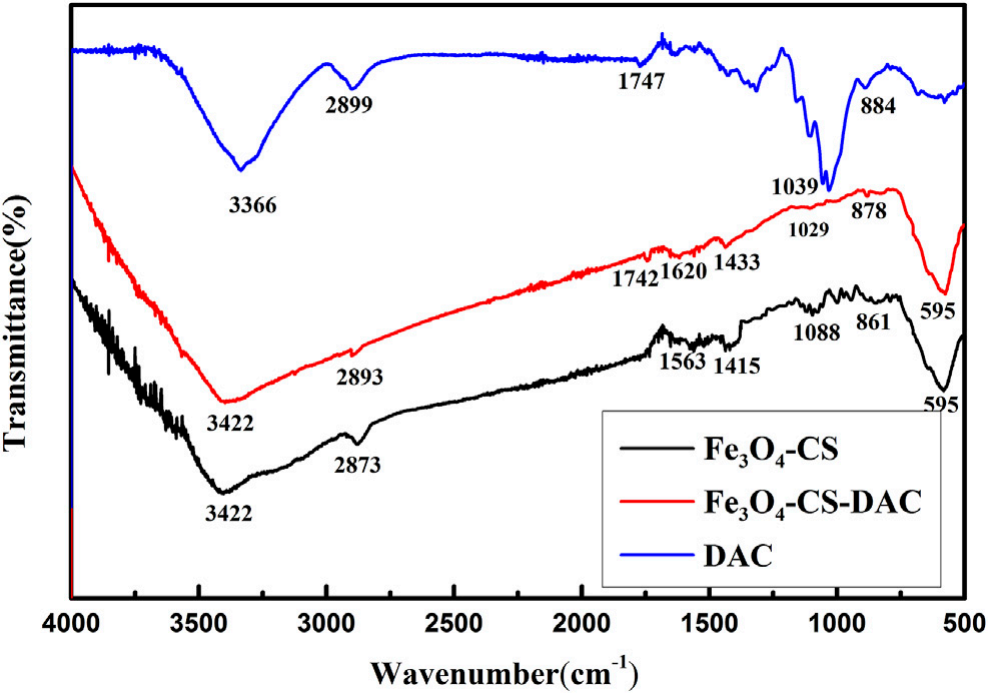
FT-IR spectrum of supports.

Figure 2 displays the FT-IR spectrum of DAC, Fe_3_O_4_-CS, and Fe_3_O_4_-CS-DAC. In the DAC spectrum, the infrared image of DAC shows the characteristic absorption peaks of the aldehyde group (–CHO) at 1,747 and 884 cm^−1^. The former is assigned to the C=O stretching of free aldehyde and the latter to the hemiacetal structure (Li et al., 2011; Yang et al., 2019). In the Fe_3_O_4_-CS spectrum, 595 cm^−1^ belongs to the characteristic peak of Fe_3_O_4_, and the spectra at 2,873, 1,563, 1,415, and 1,088 cm^−1^ are characteristic peaks of CS (Qiu et al., 2019; Suo et al., 2019). Those confirm that CS is successfully coated on Fe_3_O_4_. In the Fe_3_O_4_-CS-DAC spectrum, it contains the characteristic peak of Fe_3_O_4_. The absorption peak at 1,620 cm^−1^ is considered to be the C=N functional group formed by the Schiff base reaction between the aldehyde group of DAC and the amino group of CS (Qiu et al., 2021). At the same time, the absorption peaks at 1742 and 878 cm^−1^ confirm that there are still aldehyde groups on the surface of the nanoparticles, which can be further covalently bound to CRL.

**FIGURE 3:**
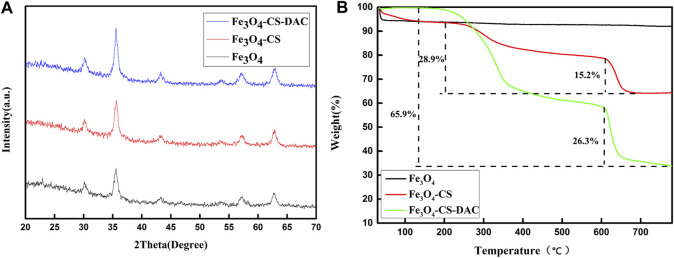
X-ray diffraction characterization **(A)** and thermogravimetric analysis curve **(B)** of support.

XRD characterization can determine the crystal structure of the material. Figure 3A shows the XRD characterization of Fe_3_O_4_, Fe_3_O_4_-CS, and Fe_3_O_4_-CS-DAC nanoparticles. The peaks appearing at 2θ = 32.6°, 35.5°, 43.2°, 53.5°, 57.0°, and 63.0° are consistent with the characteristic peaks of the crystal structure of magnetic nanoparticles Fe_3_O_4_ (Nguyen et al., 2017), which proves that the crystal structure of Fe_3_O_4_ is not damaged.

The weight loss of Fe_3_O_4_, Fe_3_O_4_-CS, and Fe_3_O_4_-CS-DAC are shown in Figure 3B. The weight of supports dropping before 150°C may be mainly due to the loss of water adsorbed on the nanoparticles. The weight loss after 150°C may have been caused by CS and DAC on the surface of the supports. Therefore, the results can indicate that DAC modified by CS was successfully coated on the surface of Fe_3_O_4_.

**FIGURE 4:**
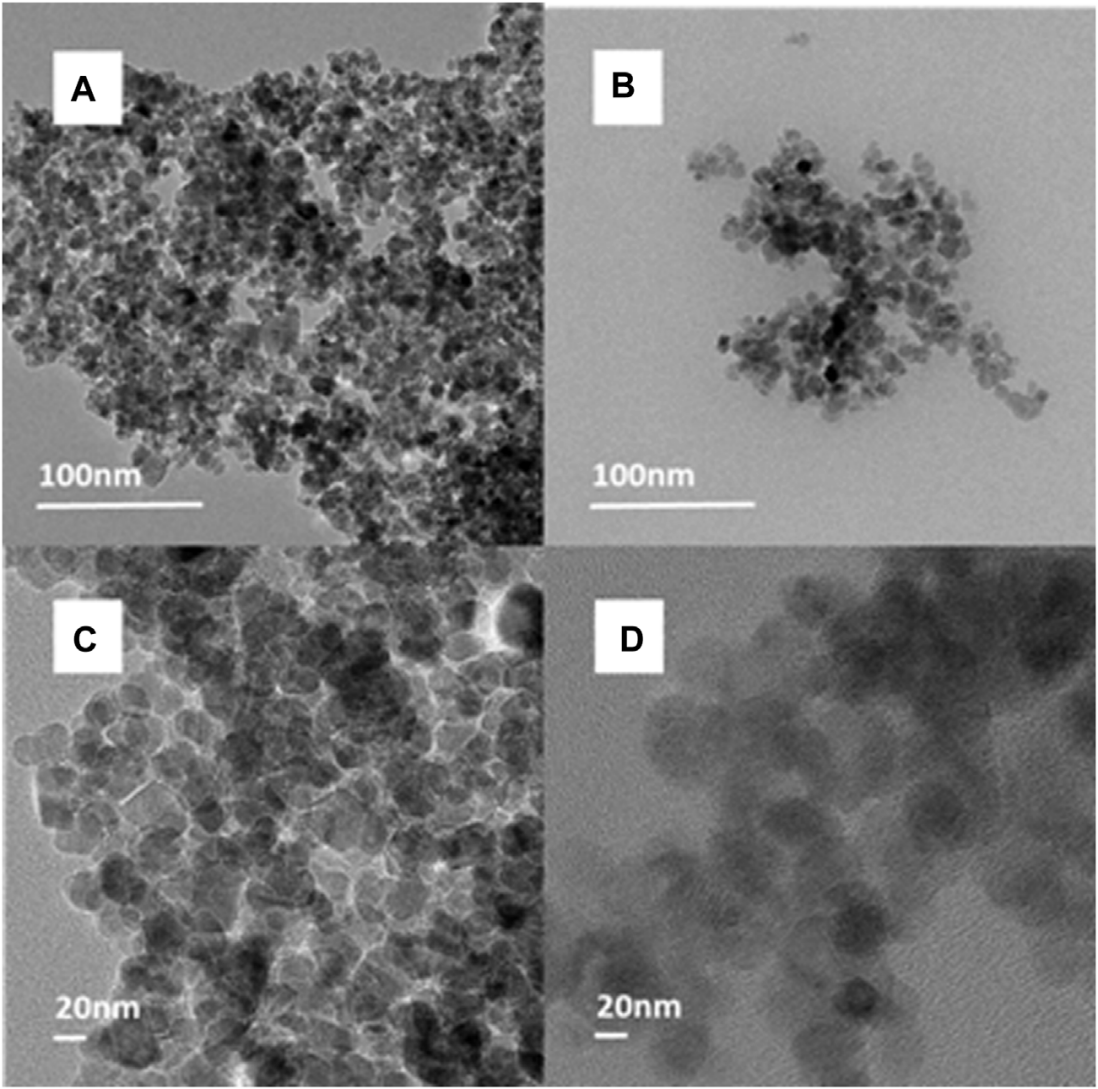
TEM images of **(A,C)** Fe_3_O_4_-CS and **(B,D)** Fe_3_O_4_-CS-DAC.

Figure 4 shows the TEM images of Fe_3_O_4_-CS and Fe_3_O_4_-CS-DAC. From Figures 4A and C, it can be seen that the boundary structure becomes blurred owing to the CS covering the surface of Fe_3_O_4_. From Figures 4B and D, it can be seen that after introducing macromolecule DAC with abundant aldehyde and hydroxy groups, which could prevent particle agglomeration through electrostatic repulsive force between particles, the agglomeration phenomenon of the support is reduced. The surface of the support is further blurred, and the particle size is significantly larger than that of Fe_3_O_4_-CS; those phenomena indicate the successful connection of DAC.

### Results of *Candida rugosa* Lipase Immobilization

Table 1 shows the effect of different surfactants on the immobilization of CRL to Fe_3_O_4_-CS-DAC and the effect of using SDS as surfactants to immobilize CRL onto other supports. The results show that the immobilization efficiency of physical adsorption (Fe_3_O_4_-CS) and covalent cross-linking (Fe_3_O_4_-CS-GA and Fe_3_O_4_-CS-DAC) do not change; obviously, those supports all obtain high enzyme loading. The high loading is because organic–inorganic nanocomposites may provide more interaction sites for the immobilization of CRL. Among those immobilized CRLs, the activity of Fe_3_O_4_-CS-DAC-CRL is significantly improved. Compared with traditional cross-linking agents such as GA, DAC with good biocompatibility can effectively reduce the mass transfer limitation of the substrate and improve the affinity of the immobilized CRL to the substrate (Nguyen et al., 2017). Wong et al. (2020) coated the surface of Fe_3_O_4_ with SiO_2_ and modified it with 3-aminopropyltri ethoxysilane, and then they used GA as the cross-linking agent to covalently immobilize CRL, while the loading capacity was only 14.7 mg/g. Ji et al. (2021) reported a one-pot strategy to achieve the successful encapsulation of CRL and Fe_3_O_4_ nanoparticles into ZIF-8. The immobilized CRL only had 20.9 mg/g enzyme loading. Adikwu et al. (2021) used GA as the cross-linking agent to immobilize CRL onto the amino-functionalized SiO_2_/Fe_3_O_4_/GO ternary composite, but the enzyme loading was only 24.7 mg/g, and the specific activity was only 65.2 U/g. Compared with that in the above documents, it is worth mentioned that higher enzyme loading and activity could be obtained using the novel support (Fe_3_O_4_-CS-DAC).

**TABLE 1:** Immobilization efficiency and catalytic activity of *Candida rugosa* lipase (CRL).

Support	Enzyme immobilize		Enzyme assay	
	Immobilization efficiency (%)	CRL loading (mg/g)	Expressed activity (U/g)	Specific activity (U/g)
Fe_3_O_4_-CS	59.1	57.5	15.2	264.3
Fe_3_O_4_-CS-GA	56.4	53.9	25.4	417.2
Fe_3_O_4_-CS-DAC	57.8	55.2	42.8	775.4
Fe_3_O_4_-CS-DAC-Triton X-100	58.7	58.2	42.9	737.1
Fe_3_O_4_-CS-DAC-Tween 80	58.3	57.7	41.4	717.5
Fe_3_O_4_-CS-DAC-SDS	61.6	60.9	48.7	799.7
Fe_3_O_4_-CS-SDS	58.2	57.1	25.6	448.3
Fe_3_O_4_-CS-GA-SDS	57.1	56.5	36.8	651.3

The protein content of CRL is 264.6 mg/g, and the specific activity of free CRL is 460.0 U/g.

The results show that the immobilization efficiency is slightly improved after adding three types of surfactants and the anionic surfactant SDS has the best effect especially. The results also show that the effect of using SDS as the surfactant to immobilize CRL onto different supports change slightly. However, the specific activities of immobilized CRLs are all improved to a certain extent, and Fe_3_O_4_-CS-DAC-SDS shows the best immobilization of CRL. Compared with nonionic surfactants, anionic surfactants have a stronger hydrophobic effect (Holmberg et al., 2018; Ozyilmaz et al., 2020). Zhang et al. (2018) employed molecular docking to provide an insight into the interactions of CRL and SDS. They found that the principal binding region of SDS and CRL is close to the hydrophobic force–dominated cavity, which can keep the active conformation of CRL. In addition, SDS changes the microenvironment polarity of CRL and combines with CRL through hydrophobic force, hydrogen bonding force. and electrostatic force; thus, it will partially or completely unfold the tertiary structure of CRL. These effects transfer CRL from the relatively closed form to the more open form and increases its activity (Biasutti et al., 2008; Gabriele et al., 2018; Katiyar et al., 2021).

### Influence of pH and Temperature on *Candida rugosa* Lipase Activity

**FIGURE 5:**
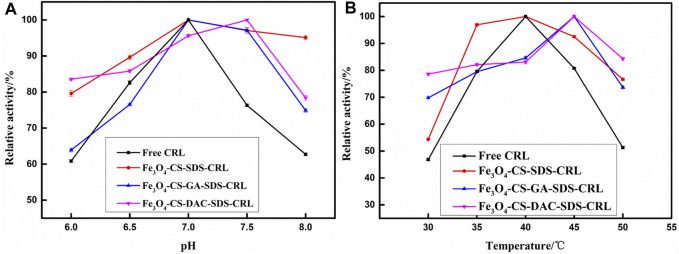
Effect of pH **(A)** and temperature **(B)** on free and immobilized CRL activity.

Figure 5A shows the effect of pH (6.0–8.0) on the activity of free and immobilized CRL at 40°C. The sensitivity of immobilized CRL to pH is slightly reduced compared with that of free CRL. Optimal pH value and better acid/alkaline resistance may indicate that DAC or GA provides multiple attachment sites for CRL to maintain its conformation and increase its resistance to environmental changes (Nguyen et al., 2017).

It can be seen from Figure 5B that the sensitivity of immobilized CRL to temperature is reduced. The optimum temperature of Fe_3_O_4_-CS-GA-SDS-CRL and Fe_3_O_4_-CS-DAC-SDS-CRL is higher than that of free CRL. The optimum temperature of immobilized CRL moves to a higher direction; however, as the temperature further increases, the structure of CRL will be destroyed, resulting in a decrease in CRL activity. In the process of immobilization, the electrostatic and hydrogen bond interaction between CRL and CS or the cross-linking agent, especially the covalent attachment of CRL with DAC-modified CS, may lead to the retention of the biologically active conformation of CRL, which increases the rigidity of the immobilized CRL and the stability of the structure of CRL (Zhang et al., 2018).

### Thermal Stability, Storage Stability, and Reusability

**FIGURE 6:**
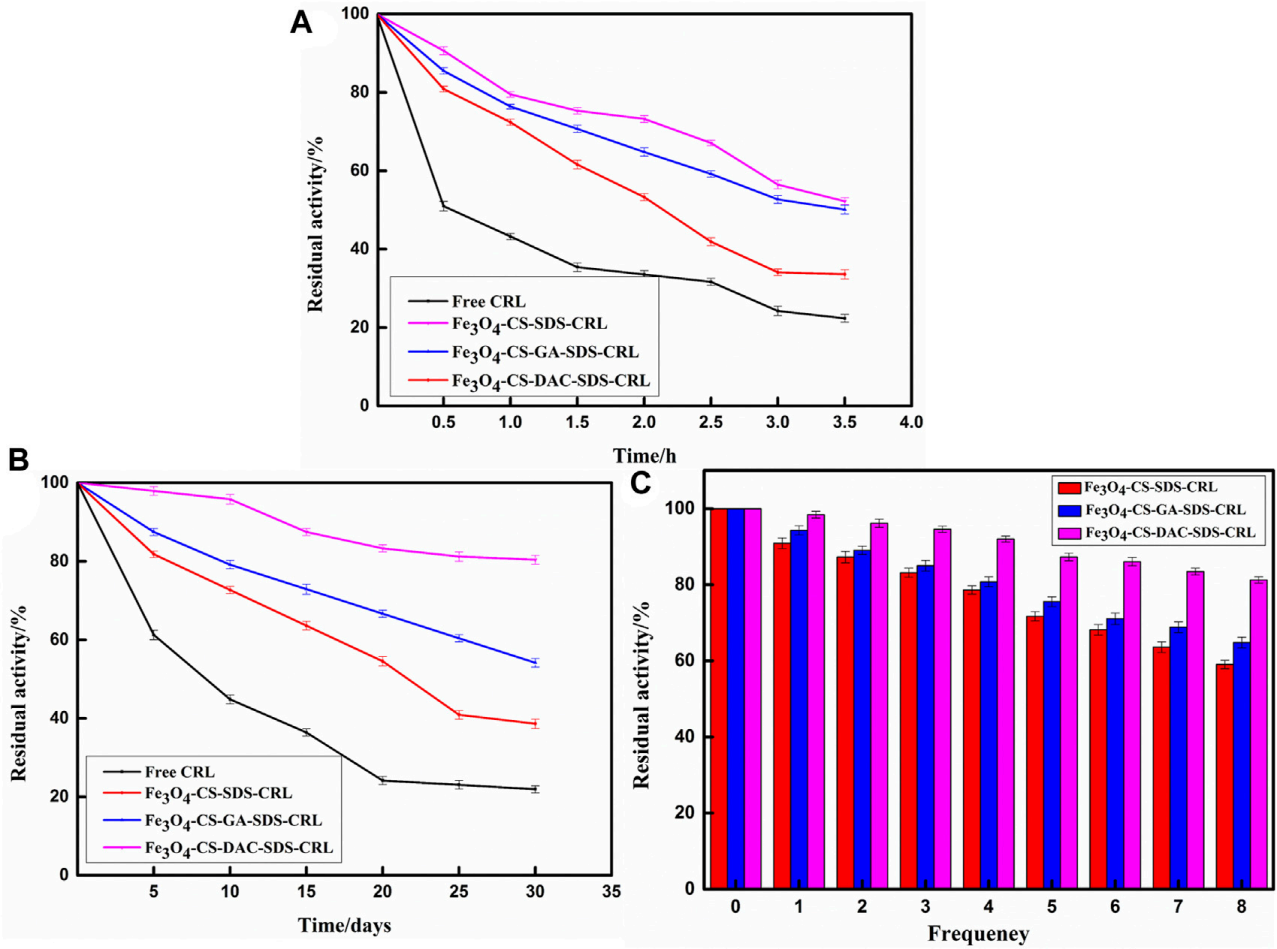
Studies on thermal stability **(A)**, storage stability **(B)**, and reusability **(C)**.

The thermal stability results are shown in Figure 6A. Although the high temperature easily destroys the secondary and tertiary structures of CRL, the activity of Fe_3_O_4_-CS-DAC-SDS-CRL is still retained at 52.3% after 3.5 h, which is much higher than that of free CRL and other immobilized CRLs. DAC has a similar chemical structure to CS, so the CS composite material with DAC has better biocompatibility. At the same time, DAC is rich in aldehyde group sites, which can increase the rigid structure and improve the thermal stability of immobilized CRL. Therefore, the introduction of DAC increases the stability of CRL to structural changes and improves the thermal stability of Fe_3_O_4_-CS-DAC-SDS-CRL (Qiu et al., 2019).

Figure 6B shows the effect of storage time on the activities of free CRL and different immobilized CRL. It can be seen that after immobilization, the support has a certain protective effect on CRL. After 30 days, Fe_3_O_4_-CS-DAC-SDS-CRL retained about 80% of the initial activity, which is much higher than those of other immobilized CRLs and free CRL. It may be that DAC as the cross-linking agent can form the stable C=N bond by Schiff base reaction, and the multiple interactions including van der Waals force, hydrogen bond, and electrostatic force between CRL and the support might be enhanced with the incorporation of DAC; therefore, the storage stability of immobilized CRL could be improved (Rashid et al., 2018).

As showed in Figure 6C, Fe_3_O_4_-CS-DAC-SDS-CRL still retains 81% activity after eight operation times, which is higher than those of other immobilized CRLs. It may be attributed to the reason that the interaction between CRL and Fe_3_O_4_-CS-DAC is more stable. The stability of CRL is enhanced and the leakage of CRL from the support is reduced when using DAC with good biocompatibility and multiple functions as the cross-linking agent (Para et al., 2002; Zhang et al., 2018). Compared with many methods reported in the literature, the reusability of immobilized CRL (Fe_3_O_4_-CS-DAC-SDS-CRL) showed better performance. Zhu et al. (2015) covalently fixed CRL on polyethylene imide–modified magnetic Fe_3_O_4_ using GA as the cross-linking agent, and the immobilized CRL retained only 75.0% of its initial activity after repeated use for eight times. Guo et al. (2021) used DAC as the cross-linking agent to immobilize *Rhizopus* lipase onto aminated Fe_3_O_4_ and found that the activity of immobilized lipase only retained 52.1% after six runs.

### Kinetic Parameters of Immobilized Enzyme

By drawing the Lineweaver–Burk diagram of immobilized CRL, the apparent Michaelis constant (K_m_), the maximum enzymatic reaction rate (V_max_), and the catalytic efficiency (K_m_/V_max_) can be calculated, which are listed in Table 2. The kinetic parameters show that compared with Fe_3_O_4_-CS-SDS-CRL, the introduction of DAC significantly reduces the Km value of the support. In general, the decrease in Km value means that the affinity of reaction substrate and enzyme is enhanced, which may be more favorable for the substrate to enter the active domain of enzyme. The decrease of V_max_ may be attributed to the increase of steric hindrance caused by the introduction of DAC macromolecules. In general, the V_max_/K_m_ value is increased, which is conducive to the increase of hydrolysis activity (Xia et al., 2016; Qiu et al., 2020).

**TABLE 2:** Kinetic parameters of immobilized CRL.

Sample	V_max_ (mg/ml·min)	K_m_ (mg/ml)	V_max_/K_m_
CRL	0.23	0.141	1.63
Fe_3_O_4_-CS-SDS-CRL	0.19	0.078	2.43
Fe_3_O_4_-CS-DAC-SDS-CRL	0.11	0.027	3.93

The protein content of CRL is 264.6 mg/g, and the specific activity of free CRL is 460.0 U/g.

### Circular Dichroism Analysis

Circular dichroism has been widely used in the analysis of the secondary structure of protein molecules. The secondary structure composition of free and immobilized CRL is listed in the Table 3. These results indicate that the content of α-helix and β-sheet in the three vectors has changed. The decrease of α-helix indicates that CRL opens a higher conformation and the active site of CRL is more exposed, which makes the substrate more accessible and thus increases the enzymatic activity. The increase of β-folding could be related to the improvement of the stability of immobilized enzymes (Zhu et al., 2015; Suo et al., 2019). The β-folding ratio of CRL increased significantly after covalent fixation, which may be due to the strong interaction between CRL and the magnetic CS carrier activated by the aldehyde group, so that the structure of CRL becomes more rigidity, which is beneficial to maintaining the active conformation of CRL and enhancing the stability of CRL. This is consistent with the previous enzymological data.

**TABLE 3:** Circular dichroism analysis of immobilized CRL.

Sample	Alpha helix/%	Total beta-sheet/%	Beta-turn/%	Random coil/%
CRL	31.3	13.7	33.0	20.7
Fe_3_O_4_-CS-SDS-CRL	25.9	18.5	34.0	19.9
Fe_3_O_4_-CS-GA-SDS-CRL	23.7	22.1	34.5	19.1
Fe_3_O_4_-CS-DAC-SDS-CRL	21.7	21.5	35.2	18.3

## Conclusion

In this study, a novel magnetic organic–inorganic nanocomposite support was prepared by combining Fe_3_O_4_, CS, and DAC with good biocompatibility and abundant functional groups through Schiff base reaction; then, CRL was covalently immobilized onto the support through the multifunctional DAC, which is first applied to immobilize CRL. The addition of anionic surfactant SDS can further increase the enzyme loading and specific activity of immobilized CRL, and the immobilized CRL exhibited strong pH and temperature tolerance, good thermal stability, and storage stability. Reusability study showed that after eight operation times, the hydrolysis activity of Fe_3_O_4_-CS-DAC-SDS-CRL could still be retained at 81.3%. Studies have confirmed that the use of macromolecular cross-linking agents and the addition of suitable surfactants can enhance the interaction between the supports and CRL, stabilize the active conformation of the enzymes, and thus enhance the activity and stability of CRL. The study of rationally analyzing the mechanism of changes in enzymatic properties of immobilized enzymes at the molecular level through methods using molecular simulation and spectroscopic structural characterization is currently under search.

## Data Availability

The original contributions presented in the study are included in the article/Supplementary Material, and further inquiries can be directed to the corresponding authors.

## References

[B1] Adikwu G. J. Wahab R. A. Mahat N. A. (2021). Ternary Biogenic Silica/magnetite/graphene Oxide Composite for the Hyperactivation of *Candida Rugosa* Lipase in the Esterification Production of Ethyl Valerate. Enzyme Microb. Technol. 148, 109807. 10.1016/j.enzmictec.2021.109807 34116744

[B3] Barbosa O. Torres R. Ortiz C. Berenguer-Murcia Á. Rodrigues R. C. Fernandez-Lafuente R. (2013). Heterofunctional Supports in Enzyme Immobilization: From Traditional Immobilization Protocols to Opportunities in Tuning Enzyme Properties. Biomacromolecules 14 (8), 2433–2462. 10.1021/bm400762h 23822160

[B4] Barriuso J. Vaquero M. E. Prieto A. Martínez M. J. (2016). Structural Traits and Catalytic Versatility of the Lipases from the *Candida Rugosa*-like Family: A Review. Biotechnol. Adv. 34 (5), 874–885. 10.1016/j.biotechadv.2016.05.004 27188926

[B6] Betancor L. López-Gallego F. Hidalgo A. Alonso-Morales N. Mateo G. D.-O. C. Fernández-Lafuente R. (2006). Different Mechanisms of Protein Immobilization on Glutaraldehyde Activated Supports: Effect of Support Activation and Immobilization Conditions. Enzyme Microb. Technol. 39 (4), 877–882. 10.1016/j.enzmictec.2006.01.014

[B7] Biasutti M. A. Abuin E. B. Silber J. J. Correa N. M. Lissi E. A. (2008). Kinetics of Reactions Catalyzed by Enzymes in Solutions of Surfactants. Adv. Colloid Interface Sci. 136 (1/2), 1–24. 10.1016/j.cis.2007.07.001 17706582

[B9] Calvo M. V. Plou F. J. Ballesteros A. (1996). Effect of Surfactants on Activity and Stability of Native and Chemically Modified Lipases A and B from *Candida Rugosa* . Biocatal. Biotransformation 13 (4), 271–285. 10.3109/10242429609003605

[B11] De Oliveira U. M. F. Lima de Matos L. J. B. De Souza M. C. M. Pinheiro B. B. Dos Santos J. C. S. Gonçalves L. R. B. (2018). Effect of the Presence of Surfactants and Immobilization Conditions on Catalysts' Properties of Rhizomucor Miehei Lipase onto Chitosan. Appl. Biochem. Biotechnol. 184 (4), 1263–1285. 10.1007/s12010-017-2622-1 29019010

[B13] Faraji M. Shirani M. Rashidi-Nodeh H. (2021). The Recent Advances in Magnetic Sorbents and Their Applications. Trac-Trends Anal. Chem. 141, 116302. 10.1016/j.trac.2021.116302

[B15] Gabriele F. Spreti N. Del Giacco T. Germani R. Tiecco M. (2018). Effect of Surfactant Structure on the Superactivity of *Candida Rugosa* Lipase. Langmuir 34 (38), 11510–11517. 10.1021/acs.langmuir.8b02255 30152702

[B16] Guo H. Lei B. Yu J. Chen Y. Qian J. (2021). Immobilization of Lipase by Dialdehyde Cellulose Crosslinked Magnetic Nanoparticles. Int. J. Biol. Macromol. 185, 287–296. 10.1016/j.ijbiomac.2021.06.073 34153359

[B17] Holmberg K. (2018). Interactions between Surfactants and Hydrolytic Enzymes. Colloids Surfaces B Biointerfaces 168, 169–177. 10.1016/j.colsurfb.2017.12.002 29248277

[B18] Hu Y. Jiang X. Wu S. Jiang L. Huang H. (2013). Synthesis of Vitamin E Succinate by Interfacial Activated *Candida Rugosa* Lipase Encapsulated in Sol-Gel Materials. Chin. J. Catal. 34 (8), 1608–1616. 10.1016/S1872-2067(12)60628-7

[B19] Ismail A. R. Kashtoh H. Baek K.-H. (2021). Temperature-resistant and Solvent-Tolerant Lipases as Industrial Biocatalysts: Biotechnological Approaches and Applications. Int. J. Biol. Macromol. 187, 127–142. 10.1016/j.ijbiomac.2021.07.101 34298046

[B20] Ji Y. Wu Z. Zhang P. Qiao M. Hu Y. Shen B. (2021). Enzyme-functionalized Magnetic Framework Composite Fabricated by One-Pot Encapsulation of Lipase and Fe3O4 Nanoparticle into Metal-Organic Framework. Biochem. Eng. J. 169, 107962. 10.1016/j.bej.2021.107962

[B21] Katiyar M. Abida K. Ali A. (2021). *Candida Rugosa* Lipase Immobilization over SBA-15 to Prepare Solid Biocatalyst for Cotton Seed Oil Transesterification. Mater. Today Proc. 36 (3), 763–768. 10.1016/j.matpr.2020.06.061

[B22] Kim U.-J. Lee Y. R. Kang T. H. Choi J. W. Kimura S. Wada M. (2017). Protein Adsorption of Dialdehyde Cellulose-Crosslinked Chitosan with High Amino Group Contents. Carbohydr. Chem. 163, 34–42. 10.1016/j.carbpol.2017.01.052 28267516

[B24] Leung H.-W. (2001). Ecotoxicology of Glutaraldehyde: Review of Environmental Fate and Effects Studies. Ecotoxicol. Environ. Saf. 49 (1), 26–39. 10.1006/eesa.2000.2031 11386713

[B25] Li H. Wu B. Mu C. Lin W. (2011). Concomitant Degradation in Periodate Oxidation of Carboxymethyl Cellulose. Carbohydr. Polym. 84 (3), 881–886. 10.1016/j.carbpol.2010.12.026

[B26] Li X. Zhang C. Li S. Huang H. Hu Y. (2015). Improving Catalytic Performance of *Candida Rugosa* Lipase by Chemical Modification with Polyethylene Glycol Functional Ionic Liquids. Ind. Eng. Chem. Res. 54 (33), 8072–8079. 10.1021/acs.iecr.5b01881

[B27] Liang S. Wu X.-L. Xiong J. Zong M.-H. Lou W.-Y. (2020). Metal-organic Frameworks as Novel Matrices for Efficient Enzyme Immobilization: An Update Review. Coord. Chem. Rev. 406, 213149. 10.1016/j.ccr.2019.213149

[B28] López-Serrano P. Cao L. van Rantwijk F. Sheldon R. A. (2002). Cross-linked Enzyme Aggregates with Enhanced Activity: Application to Lipases. Biotechnol. Lett. 24 (16), 1379–1383. 10.1023/a:1019863314646

[B29] Manoel E. A. dos Santos J. C. S. Freire D. M. G. Rueda N. Fernandez-Lafuente R. (2015). Immobilization of Lipases on Hydrophobic Supports Involves the Open Form of the Enzyme. Enzyme Microb. Technol. 71, 53–57. 10.1016/j.enzmictec.2015.02.001 25765310

[B30] Mateo C. Palomo J. M. Fernandez-Lorente G. Guisan J. M. Fernandez-Lafuente R. (2007). Improvement of Enzyme Activity, Stability and Selectivity via Immobilization Techniques. Enzyme Microb. Technol. 40 (6), 1451–1463. 10.1016/j.enzmictec.2007.01.018

[B31] Matveeva V. G. Bronstein L. M. (2021). Magnetic Nanoparticle-Containing Supports as Carriers of Immobilized Enzymes: Key Factors Influencing the Biocatalyst Performance. Nanomaterials 11 (9), 2257. 10.3390/nano11092257 34578573 PMC8469579

[B32] Monier M. Wei Y. Sarhan A. A. (2010). Evaluation of the Potential of Polymeric Carriers Based on Photo-Crosslinkable Chitosan in the Formulation of Lipase from *Candida Rugosa* Immobilization. J. Mol. Catal. B Enzym. 63 (1-2), 93–101. 10.1016/j.molcatb.2009.12.015

[B33] Nguyen X. S. Zhang G. Yang X. (2017). Mesocrystalline Zn-Doped Fe3O4 Hollow Submicrospheres: Formation Mechanism and Enhanced Photo-Fenton Catalytic Performance. ACS Appl. Mat. Interfaces 9 (10), 8900–8909. 10.1021/acsami.6b16839 28233986

[B34] Nunes Y. L. de Menezes F. L. de Sousa I. G. Cavalcante A. L. G. Cavalcante F. T. T. da Silva Moreira K. (2021). Chemical and Physical Chitosan Modification for Designing Enzymatic Industrial Biocatalysts: How to Choose the Best Strategy? Int. J. Biol. Macromol. 181, 1124–1170. 10.1016/j.ijbiomac.2021.04.004 33864867

[B35] Ozyilmaz E. Eski F. (2020). Effect of Cyclic and Acyclic Surfactants on the Activity of *Candida Rugosa* Lipase. Bioprocess Biosyst. Eng. 43 (11), 2085–2093. 10.1007/s00449-020-02397-3 32601811

[B37] Para A. Karolczyk-Kostuch S. (2002). Metal Complexes of Starch Dialdehyde Dithiosemicarbazone. Carbohydr. Polym. 50 (2), 151–158. 10.1016/S0144-8617(02)00011-5

[B38] Qiao W. Zhang Z. Qian Y. Xu L. Guo H. (2022). Bacterial Laccase Immobilized on a Magnetic Dialdehyde Cellulose without Cross-Linking Agents for Decolorization. Colloids Surfaces A Physicochem. Eng. Aspects 632, 127818. 10.1016/j.colsurfa.2021.127818

[B39] Qiu X. Qin J. Xu M. Kang L. Hu Y. (2019). Organic-inorganic Nanocomposites Fabricated via Functional Ionic Liquid as the Bridging Agent for Laccase Immobilization and its Application in 2,4-dichlorophenol Removal. Colloids Surfaces B Biointerfaces 179, 260–269. 10.1016/j.colsurfb.2019.04.002 30978613

[B40] Qiu X. Wang S. Miao S. Suo H. Xu H. Hu Y. (2021). Co-immobilization of Laccase and ABTS onto Amino-Functionalized Ionic Liquid-Modified Magnetic Chitosan Nanoparticles for Pollutants Removal. J. Hazard. Mater. 401, 123353. 10.1016/j.jhazmat.2020.123353 32652421

[B41] Qiu X. Wang Y. Xue Y. Li W. Hu Y. (2020). Laccase Immobilized on Magnetic Nanoparticles Modified by Amino-Functionalized Ionic Liquid via Dialdehyde Starch for Phenolic Compounds Biodegradation. Chem. Eng. J. 391, 123564. 10.1016/j.cej.2019.123564

[B42] Rashid R. Anwar Z. Zafar M. Rashid T. Butt I. (2018). Chitosan-alginate Immobilized Lipase Based Catalytic Constructs: Development, Characterization and Potential Applications. Int. J. Biol. Macromol. 119, 992–1001. 10.1016/j.ijbiomac.2018.07.192 30081130

[B43] Rodrigues R. C. Berenguer-Murcia Á. Fernandez-Lafuente R. (2011). Coupling Chemical Modification and Immobilization to Improve the Catalytic Performance of Enzymes. Adv. Synth. Catal. 353 (13), 2216–2238. 10.1002/adsc.201100163

[B44] Ruan C.-Q. Strømme M. Lindh J. (2018). Preparation of Porous 2,3-dialdehyde Cellulose Beads Crosslinked with Chitosan and Their Application in Adsorption of Congo Red Dye. Carbohydr. Polym. 181, 200–207. 10.1016/j.carbpol.2017.10.072 29253964

[B45] Schmid R. D. Verger R. (1998). Lipases: Interfacial Enzymes with Attractive Applications. Angew. Chem. Int. Ed. Engl. 37 (12), 1608–1633. 10.1002/(SICI)1521-3773(19980703)37:12<1608::AID-ANIE1608>3.0.CO;2-V 29711530

[B46] Sharma P. R. Varma A. J. (2014). Thermal Stability of Cellulose and Their Nanoparticles: Effect of Incremental Increases in Carboxyl and Aldehyde Groups. Carbohydr. Polym. 114, 339–343. 10.1016/j.carbpol.2014.08.032 25263899

[B47] Sherawat M. Rahi R. K. Gupta V. Neelam D. DevkiSain D. (2021). Prevention and Control of Food Spoilage: An Overview (Review Article). ijpbs 11 (1), 124–130. 10.1016/j.carbpol.2014.08.03210.21276/ijpbs.2021.11.1.16

[B48] Suo H. Gao Z. Xu L. Xu C. Yu D. Xiang X. (2019). Synthesis of Functional Ionic Liquid Modified Magnetic Chitosan Nanoparticles for Porcine Pancreatic Lipase Immobilization. Mater. Sci. Eng. C Mater. Biol. Appl. 96, 356–364. 10.1016/j.msec.2018.11.041 30606543

[B49] Suo H. Xu L. Xu C. Chen H. Yu D. Gao Z. (2018). Enhancement of Catalytic Performance of Porcine Pancreatic Lipase Immobilized on Functional Ionic Liquid Modified Fe3O4-Chitosan Nanocomposites. Int. J. Biol. Macromol. 119, 624–632. 10.1016/j.ijbiomac.2018.07.187 30071225

[B51] Tamaddon F. Arab D. (2019). Urease Covalently Immobilized on Cotton-Derived Nanocellulose-Dialdehyde for Urea Detection and Urea-Based Multicomponent Synthesis of Tetrahydro-Pyrazolopyridines in Water. RSC Adv. 9 (71), 41893–41902. 10.1039/C9RA05240B 35541594 PMC9076516

[B53] Vanleeuw E. Winderickx S. Thevissen K. Lagrain B. Dusselier M. Cammue B. P. A. (2019). Substrate-specificity of *Candida Rugosa* Lipase and its Industrial Application. ACS Sustain. Chem. Eng. 7 (19), 15828–15844. 10.1021/acssuschemeng.9b03257

[B54] Varma A. J. Kulkarni M. P. (2002). Oxidation of Cellulose under Controlled Conditions. Polym. Degrad. Stab. 77 (1), 25–27. 10.1016/S0141-3910(02)00073-3

[B55] Verger R. (1997). 'Interfacial Activation' of Lipases: Facts and Artifacts. Trends Biotechnol. 15 (1), 32–38. 10.1016/S0167-7799(96)10064-0

[B56] Walker J. M. (1996). The Bicinchoninic Acid (BCA) Assay for Protein Quantitation. Methods Mol. Biol. 32, 11–14. 10.1007/978-1-60327-259-9_3 7951748

[B57] Wang J. Zhao G. Jing L. Peng X. Li Y. (2015). Facile Self-Assembly of Magnetite Nanoparticles on Three-Dimensional Graphene Oxide-Chitosan Composite for Lipase Immobilization. Biochem. Eng. J. 98, 75–83. 10.1016/j.bej.2014.11.013

[B58] Wong W. K. L. Wahab R. A. Onoja E. (2020). Chemically Modified Nanoparticles from Oil Palm Ash Silica-Coated Magnetite as Support for *Candida Rugosa* Lipase-Catalysed Hydrolysis: Kinetic and Thermodynamic Studies. Chem. Pap. 74 (4), 1253–1265. 10.1007/s11696-019-00976-7

[B59] Xia T.-T. Liu C.-Z. Hu J.-H. Guo C. (2016). Improved Performance of Immobilized Laccase on Amine-Functioned Magnetic Fe 3 O 4 Nanoparticles Modified with Polyethylenimine. Chem. Eng. J. 295, 201–206. 10.1016/j.cej.2016.03.044

[B61] Xiang X. R. Huang H. Hu Y. (2017). Research Progress on Enzyme Immobilized on Nanocomposites. Chin. J. Inorg. Chem. 33 (1), 1–15. 10.11862/CJIC.2017.016

[B62] Yang X. Chen Y. Yao S. Qian J. Guo H. Cai X. (2019). Preparation of Immobilized Lipase on Magnetic Nanoparticles Dialdehyde Starch. Carbohydr. Polym. 218, 324–332. 10.1016/j.carbpol.2019.05.012 31221337

[B63] Zhang R. Liu Y. Huang X. Xu M. Liu R. Zong W. (2018). Interaction of a Digestive Protease, Candida Rugosa Lipase, with Three Surfactants Investigated by Spectroscopy, Molecular Docking and Enzyme Activity Assay. Sci. Total Environ. 622-623, 306–315. 10.1016/j.scitotenv.2017.11.305 29220758

[B64] Zhao H. Heindel N. D. (1991). Determination of Degree of Substitution of Formyl Groups in Polyaldehyde Dextran by the Hydroxylamine Hydrochloride Method. Pharm. Res. 08 (3), 400–402. 10.1023/A:1015866104055 1711201

[B65] Zhao K. Chen B. Li C. Li X. F. Li K. B. Shen Y. H. (2018). Immobilization of Candida Rugosa Lipase on Glutaraldehyde-Activated Fe 3 O 4 @Chitosan as a Magnetically Separable Catalyst for Hydrolysis of Castor Oil. Eur. J. Lipid Sci. Technol. 120 (1), 1700373. 10.1002/ejlt.201700373

[B68] Zhu W. Li Y. Zeng F. Yin H. Wang L. Zhu H. (2015). Superparamagnetic Fe3O4nanoparticles Modified by Water-Soluble and Biocompatible Polyethylenimine for Lipase Immobilization with Physical and Chemical Mechanisms. RSC Adv. 5 (29), 23039–23045. 10.1039/c4ra15832f

[B69] Ziegler-Borowska M. Chelminiak-Dudkiewicz D. Siódmiak T. Sikora A. Wegrzynowska-Drzymalska K. Skopinska-Wisniewska J. (2017). Chitosan-collagen Coated Magnetic Nanoparticles for Lipase Immobilization-New Type of "Enzyme Friendly" Polymer Shell Crosslinking with Squaric Acid. Catalysts 7 (1), 26–14. 10.3390/catal7010026

