# Supplementary material for: Immobilization of Interfacial Activated Candida rugosa Lipase Onto Magnetic Chitosan Using Dialdehyde Cellulose as Cross-Linking Agent
Source: Front Bioeng Biotechnol. 2022 Jul 18;10:946117. doi: 10.3389/fbioe.2022.946117 (PMC9340543; doi:10.3389/fbioe.2022.946117)
Supplement: Supplementary file 1 [file DataSheet1.docx]

Supplementary Material

# Methods

## Optimization of immobilization conditions

During the optimization of immobilization conditions, CRL activity was measured at pH 7.0 and 40°C.

### pH

The carrier (Fe_3_O_4_-CS-CRL, Fe_3_O_4_-CS-GA-CRL or Fe_3_O_4_-CS-DAC-CRL, 100 mg) was sonicated into 5 mL phosphate buffer (0.2 M, pH 6.0-8.0), and then 30 mg CRL was added in the suspension. The system was shaken at 30°C for 5 h. The immobilized enzyme was separated with a magnet and washed several times with the buffer solution. The prepared immobilized CRLs were designated as (Fe_3_O_4_-CS-CRL, Fe_3_O_4_-CS-GA-CRL or Fe_3_O_4_-CS-DAC-CRL, respectively. The activity of immobilized CRLs was examined and the maximum CRL activity was defined as 100%.

### Temperature

The carrier (Fe_3_O_4_-CS-CRL, Fe_3_O_4_-CS-GA-CRL or Fe_3_O_4_-CS-DAC-CRL, 100 mg) was sonicated into 5 mL phosphate buffer (0.2 M, pH 7.0), and then 30 mg CRL was added in the suspension. The system was shaken at 25-45°C for 5 h. The immobilized enzyme was separated with a magnet and washed several times with the buffer solution. The activity of immobilized CRLs was examined and the maximum CRL activity was defined as 100%.

### Enzyme concentration

The carrier (Fe_3_O_4_-CS-CRL, Fe_3_O_4_-CS-GA-CRL or Fe_3_O_4_-CS-DAC-CRL, 100 mg) was sonicated into 5 mL mixture containing citrate buffer solution (0.1 M, pH 7.0) and then CRL solution (concentration: 20-40 mg CRL). The system was shaken at respective optimum temperature conditions for 5 h. The immobilized enzyme was separated with a magnet and washed several times with the buffer solution. The activity of immobilized CRLs was examined and the maximum CRL activity was defined as 100%.

### Optimal pH and Temperature of immobilized CRLs

To study optimal pH conditions for activity assay, free and immobilized CRLs activity were measured under different pH values (pH6.0-8.0) at 40°C. The optimum temperature values were obtained by examining the activity of free and immobilized CRLs at 30-50°C under respective optimal pH conditions. With the relative activity as an index, the maximum activity was defined as 100%.

### Determination of aldehyde group content

The amount of aldehyde groups of DAC or support was determined by the standard hydroxylamine method (Zhao et al., 1991): 0.01 g DAC or support to be tested was dissolved under stirring at room temperature for 8h in 25 mL of 25 mmol hydroxylamine hydrochloride (NH_2_OH·HCl) at initial pH of 4. Released hydrochloric acid acidifying the solution was then dosed back raising the pH to the initial one via a NaOH solution (0.1 mol/L) under stirring and measurement with a pH meter. The dialdehyde content was thus determined as:

DC (%)=(V_NaOH_×C_NaOH_)/(m_sample_/M*_w_*) ×100%

Mw-the molar weight of the DAC.

# Results

## pH

Fe_3_O_4_-CS: 7; Fe_3_O_4_-CS-GA: 6.5; Fe_3_O_4_-CS-DAC: 6.5

Fig. S1 Effect of pH on CRL immobilization

## Temperature

Fe_3_O_4_-CS: 30°C; Fe_3_O_4_-CS-GA: 40°C; Fe_3_O_4_-CS-DAC: 30°C

Fig. S2 Effect of temperature on CRL immobilization

## Enzyme concentration

Fe_3_O_4_-CS: 40 mg CRL; Fe_3_O_4_-CS-GA: 40 mg CRL; Fe_3_O_4_-CS-DAC: 40 mg CRL

Fig. S3 Effect of enzyme concentration on CRL immobilization

## Aldehyde group content

After the determination of aldehyde group content, we found that 65% of aldehyde group was retained in the support of DAC and 15% of aldehyde group was retained in the support of Fe_3_O_4_-CS-DAC.

## Characterizations


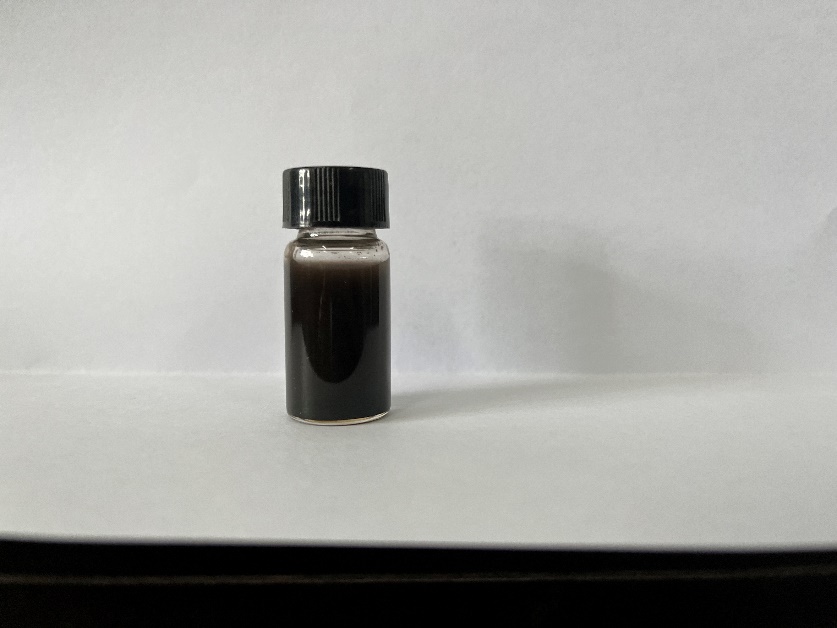

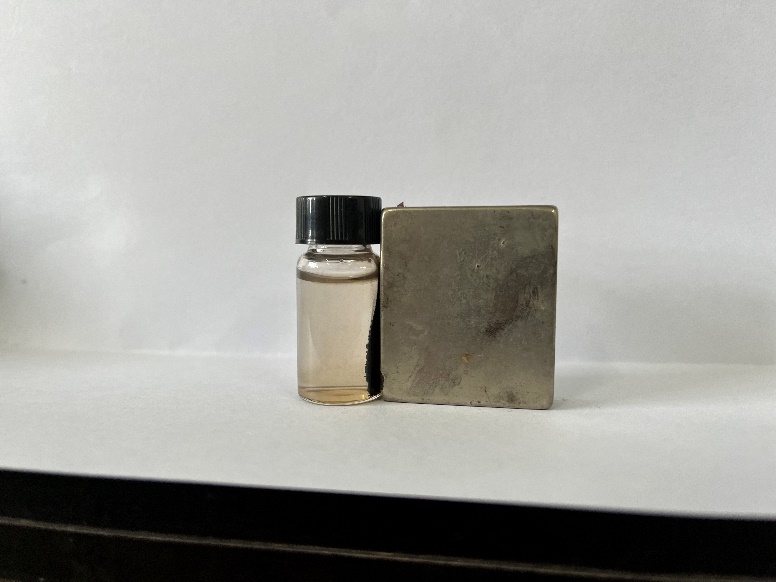


Fig.S4 Immobilized CRL magnetic nanoparticles attracted by a magnet

It can be seen from the figure that the prepared immobilized CRL (Fe_3_O_4_-CS-DAC-CRL) shows good magnetic response. This also shows that the immobilized CRL can be easily separated from the reaction system by the magnetic, which will greatly improves the reusability of the immobilized enzyme.

***References***

Zhao H., Heindel N. D. (1991). Determination of degree of substitution of formyl groups in polyaldehyde dextran by the hydroxylamine hydrochloride method. *Pharmaceutical Research*, 8(3): 400-402.

Zhao K., Chen B., Li C., Li X. F., Li K. B., Shen Y. H. (2018). Immobilization of Candida rugosa Lipase on Glutaraldehyde-Activated Fe_3_O_4_@Chitosan as a Magnetically Separable Catalyst for Hydrolysis of Castor Oil. *European Journal of Lipid Science and Technology*, 120(1).
